# Retrospective analysis of predictive factors for AVF dysfunction in patients undergoing MHD

**DOI:** 10.1097/MD.0000000000037737

**Published:** 2024-04-19

**Authors:** Liqin Wang, Yanna Yang, Qianqian Zhao

**Affiliations:** a Hemodialysis Center, Hefei Third Clinical College (Hefei Third People’s Hospital), Anhui Medical University, Hefei, China; b Department of Nephrology, Hefei Third Clinical College (Hefei Third People’s Hospital), Anhui Medical University, Hefei, China.

**Keywords:** autologous arteriovenous fistula, hemodialysis, internal validation, nomogram, predictive model

## Abstract

To construct an early clinical prediction model for AVF dysfunction in patients undergoing Maintenance Hemodialysis (MHD) and perform internal and external verifications. We retrospectively examined clinical data from 150 patients diagnosed with MHD at Hefei Third People’s Hospital from January 2014 to June 2023. Depending on arteriovenous fistula (AVF) functionality, patients were categorized into dysfunctional (n = 62) and functional (n = 88) cohorts. Using the least absolute shrinkage and selection operator(LASSO) regression model, variables potentially influencing AVF functionality were filtered using selected variables that underwent multifactorial logistic regression analysis. The Nomogram model was constructed using the R software, and the Area Under Curve(AUC) value was calculated. The model’s accuracy was appraised through the calibration curve and Hosmer–Lemeshow test, with the model undergoing internal validation using the bootstrap method. There were 11 factors exhibiting differences between the group of patients with AVF dysfunction and the group with normal AVF function, including age, sex, course of renal failure, diabetes, hyperlipidemia, Platelet count (PLT), Calcium (Ca), Phosphorus, D-dimer (D-D), Fibrinogen (Fib), and Anastomotic width. These identified factors are included as candidate predictive variables in the LASSO regression analysis. LASSO regression identified age, sex, diabetes, hyperlipidemia, anastomotic diameter, blood phosphorus, and serum D-D levels as 7 predictive factors. Unconditional binary logistic regression analysis revealed that advanced age (OR = 4.358, 95% CI: 1.454–13.062), diabetes (OR = 4.158, 95% CI: 1.243–13.907), hyperlipidemia (OR = 3.651, 95% CI: 1.066–12.499), D-D (OR = 1.311, 95% CI: 1.063–1.616), and hyperphosphatemia (OR = 4.986, 95% CI: 2.513–9.892) emerged as independent risk factors for AVF dysfunction in MHD patients. The AUC of the predictive model was 0.934 (95% CI: 0.897–0.971). The Hosmer-Lemeshow test showed high consistency between the model’s predictive results and actual clinical observations (χ^2^ = 1.553, *P* = .092). Internal validation revealed an AUC of 0.911 (95% CI: 0.866–0.956), with the Calibration calibration curve nearing the ideal curve. Advanced age, coexisting diabetes, hyperlipidemia, blood D-D levels, and hyperphosphatemia are independent risk factors for AVF dysfunction in patients undergoing MHD.

## 1. Introduction

Hemodialysis is a pivotal therapeutic intervention for chronic renal failure and acute kidney injury.^[1]^ Among these methodologies, the arteriovenous fistula (AVF) remains the principal vascular access route for patients undergoing maintenance hemodialysis (MHD),^[2,3]^ showing considerable efficacy among MHD patients.^[4]^ However, dysfunctional AVFs compromise therapeutic outcomes and quality of life and pose risks for grave complications, including infections, arterial aneurysms, bleeding, and thrombosis.^[5]^ Hence, investigating the risk models associated with AVF dysfunction is of paramount clinical relevance.

Research on AVF dysfunction has predominantly focused on etiological factors and functional state assessments.^[6]^ Nevertheless, since myriad factors influence the intricate AVF formation process, establishing an all-encompassing and precise risk model remains challenging. This study aimed to construct a risk model that holistically considered patient variability, vascular lesion severity, surgical techniques, and hematological indicators. The overarching objective is to equip clinicians with a more scientifically grounded and individualized treatment strategy.

In this investigation, our departure points were generic clinical characteristics and common blood parameters to develop a probability model for predicting AVF dysfunction. Specifically, we will amass fundamental data, including patient age, sex, body mass index (BMI), and diabetic and hypertensive histories, as well as hematological indices such as hemoglobin levels, white blood cell counts, and platelet counts, as foundational inputs for our risk model. Statistical analysis of these metrics will lead to the formulation of a model that gauges the likelihood of AVF dysfunction. Finally, we intended to validate this model by juxtaposing it with real clinical data to assess its predictive accuracy and reliability.

The significance of this research is 2-fold: On the one hand, by establishing an effective risk model, physicians can preemptively detect potential AVF dysfunctions, thereby initiating preventative measures. Furthermore, we introduce a novel paradigm and methodology for clinicians to enhance managing and treating patients undergoing MHD.

## 2. Methods

### 2.1. Study population and design

We retrospectively analyzed 150 patients with MHD admitted to Hefei Third People’s Hospital between January 2014 and June 2023. Age ranged from 23 to 88 years, with a mean age of (61.5 ± 14.3) years. Of the participants, 107 were male, and 43 were female. Primary pathologies included primary glomerulonephritis in 16 patients, diabetic nephropathy in 40, hypertensive nephropathy in 85, and other causes in 9. This research received ethical approval from the hospital’s medical ethics committee (Ref. No. 2023LLWL015), and informed consent was obtained from all patients.

### 2.2. Criteria for selection

Inclusion criteria: Age of 18 years or older. AVF preoperative imaging assessment for upper limb artery and vein without narrowing, calcification, or other anomalies. Exclusive use of AVF as vascular access for hemodialysis. Duration of hemodialysis via AVF of at least 6 months. All AVF surgeries were collectively performed by the same group of nephrologists. The exclusion criteria: Presence of significant coagulation dysfunction. Concurrent Thrombotic Disorders. Prolonged administration of anticoagulant and antiplatelet agents. Inadequate maturation of the AVF within 12 weeks post-procedure. Prior vascular intervention for AVF. Coexisting Malignancies.

### 2.3. AVF functional assessment: methodology and criteria

Six months after stable dialysis, all patients underwent functional AVF evaluation. The assessment employed a Philips EPIQ 7C color Doppler ultrasonographic diagnostic system utilizing a linear-array probe with a frequency of 10 MHz. Patients were instructed to lie supine, extend, and fully expose the examination area. A handheld probe that lightly pressed the skin was applied perpendicularly over the vein. The initial step was a two-dimensional measurement of the AVF anastomotic diameter, followed by an examination of the inflow artery and outflow vein trajectory, lumen size, and internal and external echoes within the lumen to discern the presence of thrombosis, vascular calcification, or stenosis. Concurrently, pulsed Doppler is used to detect the blood flow spectrum and flow velocity indicators, and the system autonomously computes the intravascular blood flow volume. If the fistula inlet flow consistently registered < 600 mL/s, once reasons such as puncture failure, improper needle tip placement, local hematoma, or dialysis hypotension were ruled out, the patient was diagnosed with AVF dysfunction.^[7]^

### 2.4. Observational indicators

General patient clinical data were retrieved from electronic medical records, outpatient logs, and telephone follow-ups. These included age, sex, BMI, primary disease causing renal failure, and duration of renal insufficiency. The primary laboratory indicators, collected 6 months after achieving stable dialysis, included common hematological parameters such as complete blood count, coagulation profile, blood electrolytes, and parathyroid hormone levels. The surgical data gathered included preoperative cephalic vein diameter, radial artery diameter, and anastomosis diameter.

### 2.5. Statistical analysis

Data processing was performed using the IBM SPSS software package (version 26.0). The normality of data distribution was tested using the Kolmogorov-Smirnov method; data were deemed normally distributed when *P* ≥ .05. Data following a normal distribution is presented as (x̅±s), and the Student *t* test was employed for statistical analysis between 2 independent samples. Non-normally distributed data were described as [M(P25, P75)] and analyzed using the Mann–Whitney *U* test. Categorical data were statistically described using proportions or ratios and analyzed with the χ^2^ test. The least absolute shrinkage and selection operator (LASSO) regression model was used to select predictive factors. The identified factors were then incorporated into a binary logistic regression model to analyze the independent predictors of AVF dysfunction, calculating the odds ratio (OR) and 95% confidence interval (CI). A Nomogram model was constructed using R 4.0.2, and its accuracy was assessed through calibration curves and the Hosmer–Lemeshow test, with internal validation achieved using the bootstrap method. Differences were considered statistically significant at *P* < .05.

## 3. Results

### 3.1. Baseline data

After the maturation of the AVF in all patients, they were followed up for a period ranging from 6 months to 5 years, with a median follow-up time of 35 months. Based on the functional status of the AVF, 150 patients with MHD were divided into a dysfunction group (n = 62) and a normal function group (n = 88). A comparative analysis of both groups in terms of general clinical data, hematological indicators, and surgical indicators revealed no statistically significant differences in terms of the primary etiology of the disease, BMI, albumin, parathyroid hormone, preoperative cephalic vein, and radial artery diameters (all *P* > .05). However, differences in age, sex, course of renal failure, diabetes, hyperlipidemia, PLT, blood calcium, blood phosphorus, D-D, Fib, and anastomosis width between the groups were statistically significant (all *P* < .05). Refer to Table 1.

**Table 1:** Baseline information for the AVF dysfunction and normal function groups.

Variable	Dysfunction group (n = 62)	Normal function group (n = 88)	*Z/t/χ^2^* value	*P* value
Age [yr, n(%)]			29.572	<.001
≥60	51 (82.3)	33 (37.5)		
˂60	11 (17.7)	55 (62.5)		
Gender [n (%)]			9.009	.003
Male	36 (58.1)	71 (80.7)		
Female	26 (41.9)	17 (19.3)		
Course of renal failure (y, x̅±s)	4.5 ± 0.4	4.6 ± 0.4	2.109	.037
BMI (kg/m^2^, x̅±s)	24.3 ± 4.5	24.8 ± 4.2	0.662	.509
Diabetes [n(%)]	37 (59.7)	28 (31.8)	11.497	.001
PLT (×10^9^/L, x̅±s)	219.4 ± 80.2	187.6 ± 71.2	−2.558	.012
ALB (g/L, x̅±s)	37.3 ± 5.3	36.9 ± 5.3	−0.461	.645
TC (mmol/L, x̅±s)	4.8 ± 1.3	3.9 ± 1.2	−4.336	*P*<.001
Phosphorus (mmol/L, x̅±s)	3.7 ± 1.3	2.1 ± 0.7	−9.669	*P*<.001
Ca (mmol/L, x̅±s)	2.8 ± 0.5	1.9 ± 0.3	−14.227	*P*<.001
D-D [mg/L, M(P25, P75)]	10.4 (8.1, 12.9)	8.4 (6.0, 10.7)	−4.057	*P*<.001
Fib [mg/L, M̅(P25, P75)]	5.6 (4.3, 7.2)	3.6 (2.4, 4.5)	−6.440	*P*<.001
PTH (pg/mL, x̅±s)	54.0 ± 7.9	54.0 ± 8.7	−0.034	.973
Cephalic vein diameter (cm, x̅±s)	2.2 ± 0.5	2.3 ± 0.6	1.060	.291
Radial artery diameter (cm, x̅±s)	2.3 ± 0.6	2.3 ± 0.6	0.300	.765
Anastomotic width (cm, x̅±s)	0.3 ± 0.2	0.5 ± 0.3	6.110	<.001
Initial disease [n (%)]			0.159	.984
Glomerular disease	7 (11.3)	9 (10.2)		
Diabetic nephropathy	17 (27.4)	23 (26.1)		
Hypertensive nephropathy	34 (54.8)	51 (58.0)		
Other	4 (6.5)	5 (5.7)		

Values are presented as number (%) or mean (±SD).

ALB = Albumin, AVF = arteriovenous fistula, BMI = body mass index, Ca = calcium, D-D = D-dimer, Fib = fibrinogen, PLT = Platelet count, PTH = parathyroid hormone, TC = total cholesterol.

### 3.2. Selection of predictive factors

**Figure 1.**
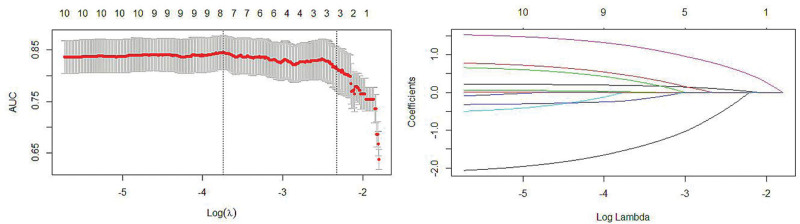
Characteristic variable selection based on LASSO regression.

Eleven factors displayed differences between the AVF dysfunction and normal function groups: age, sex, course of renal failure, diabetes, hyperlipidemia, PLT, Ca, phosphorus, D-D, Fib, and anastomotic width. These positive indicators were incorporated as candidate predictors in the LASSO regression analysis. Elderly age, female sex, diabetes, hyperlipidemia, anastomotic width, Phosphorus, and D-D, a total of 7 factors, as predictors of AVF dysfunction (Fig. 1).

### 3.3. Multivariate analysis of AVF dysfunction

Using the 7 predictive factors selected from the LASSO regression analysis (elderly, female, diabetes, hyperlipidemia, D-D, phosphorus, and anastomotic width) as independent variables and the occurrence of AVF dysfunction as the dependent variable (no dysfunction = 0, dysfunction = 1), a binary logistic regression analysis was conducted. The assignment of the independent variable values is presented in Table 2. The analysis was optimized using a stepwise backward-fitting method. The results indicated that advanced age (OR = 4.358, 95% CI: 1.454–13.062), diabetes (OR = 4.158, 95% CI: 1.243–13.907), hyperlipidemia (OR = 3.651, 95% CI: 1.066–12.499), D-D (OR = 1.311, 95% CI: 1.063–1.616), and hyperphosphatemia (OR = 4.986, 95% CI: 2.513–9.892) are independent risk factors for AVF dysfunction (all *P* < .05) (Table 3).

**Table 2:** Assignment method of independent variables in logistic regression analysis.

Independent variable	Assignment methods
Age (yr)	<60 = 0, ≥60 = 1
Gender	Male = 0, Female = 1
Anastomotic width (cm)	Measured value
Diabetes	No = 0, Yes = 1
Hyperlipidemia	No = 0, Yes = 1
D-D (mg/L)	Measured value
Phosphorus (mmol/L)	Measured value

D-D = D-dimer.

**Table 3:** Multivariate logistic regression analysis of the occurrence of AVF dysfunction.

Variable	*Β* value	SE	Wald *χ*^2^	*P* value	OR value	95% CI
Elder	1.472	0.560	6.910	.009	4.358	1.454–13.062
Diabetes	1.425	0.616	5.350	.021	4.158	1.243–13.907
Hyperlipidemia	1.295	0.628	4.252	.039	3.651	1.066–12.499
D-D	0.271	0.107	6.427	.011	1.311	1.063–1.616
Phosphorus	1.607	0.350	21.126	<.001	4.986	2.513–9.892
Constant	-8.785	1.618	29.470	<.001		

AVF = arteriovenous fistula, D-D = D-dimer.

### 3.4. Establishing a predictive model for AVF dysfunction

**Figure 2.**
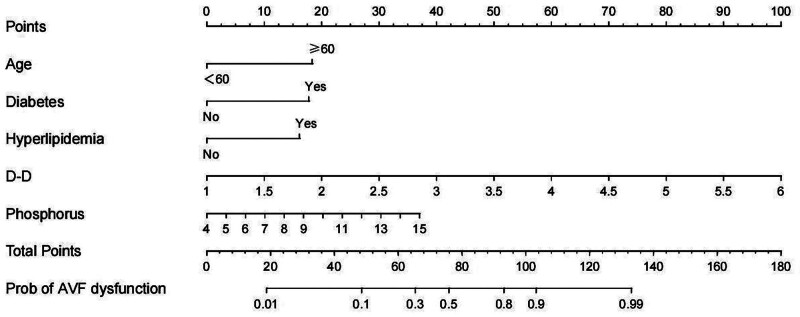
Nomogram plot of AVF malfunction prediction model. AVF = arteriovenous fistula.

**Figure 3.**
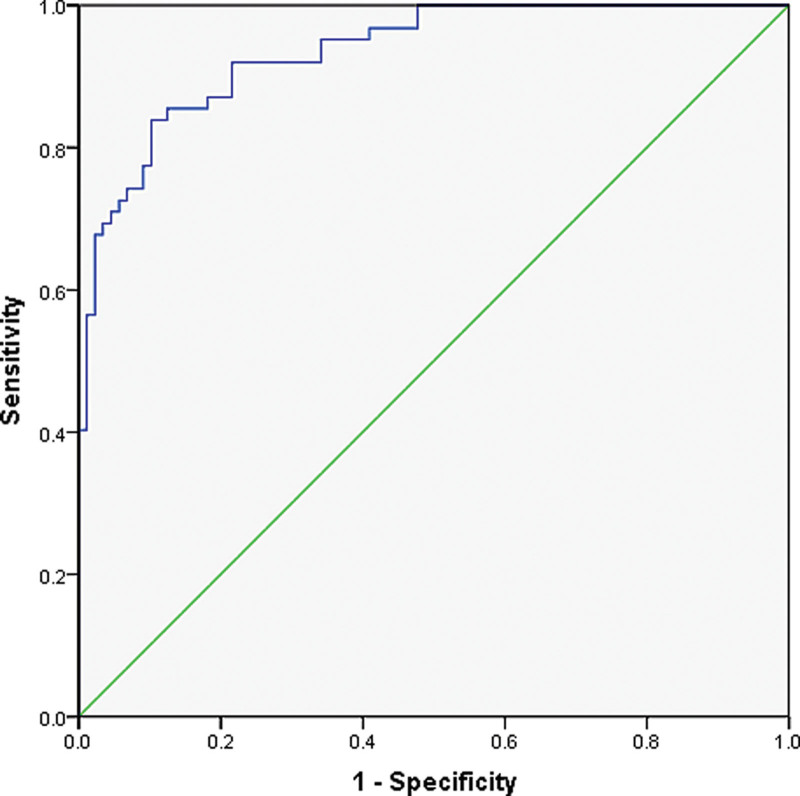
Diagnostic efficacy of predictive models.

Using the independent influencing factors from the aforementioned multivariate analysis (Elder, Diabetes, Hyperlipidemia, D-D, Phosphorus), the regression equation for the predictive model was established as follows: Logit(P) = 1.472 × Age + 1.425 × Diabetes + 1.295 × Hyperlipidemia + 0.271 × D-D + 1.607 × Phosphorus-8.785. A nomogram was constructed based on this regression equation (Fig. 2). The area under the curve (AUC) of this model was 0.934 (95% CI: 0.897–0.971), with a sensitivity and specificity of 0.839 and 0.898, respectively. Refer to Figure 3.

### 3.5. Assessing the consistency of the predictive model

**Figure 4.**
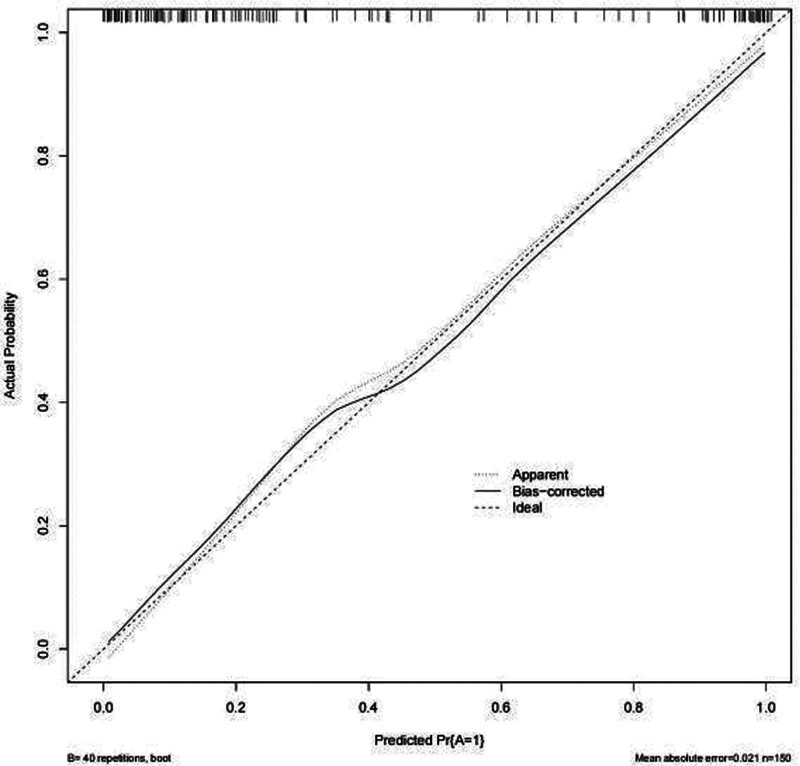
Calibration calibration curve for AVF malfunction prediction model. AVF = arteriovenous fistula.

The Hosmer-Lemeshow test showed no statistically significant difference between the predicted incidence rate of AVF dysfunction and the actual probability of occurrence (χ^2^ = 1.553, *P* = .092). This suggests that the model passed the Hosmer-Lemeshow test, indicating no pronounced difference between the predicted and actual values and demonstrating a good model fit. A Calibration curve for the predictive model was plotted, showing strong congruence between the predicted and actual curves. This suggests that the model provides a consistent and accurate prediction of AVF dysfunction risk (Fig. 4).

### 3.6. Internal validation of the AVF dysfunction risk prediction model

**Figure 5.**
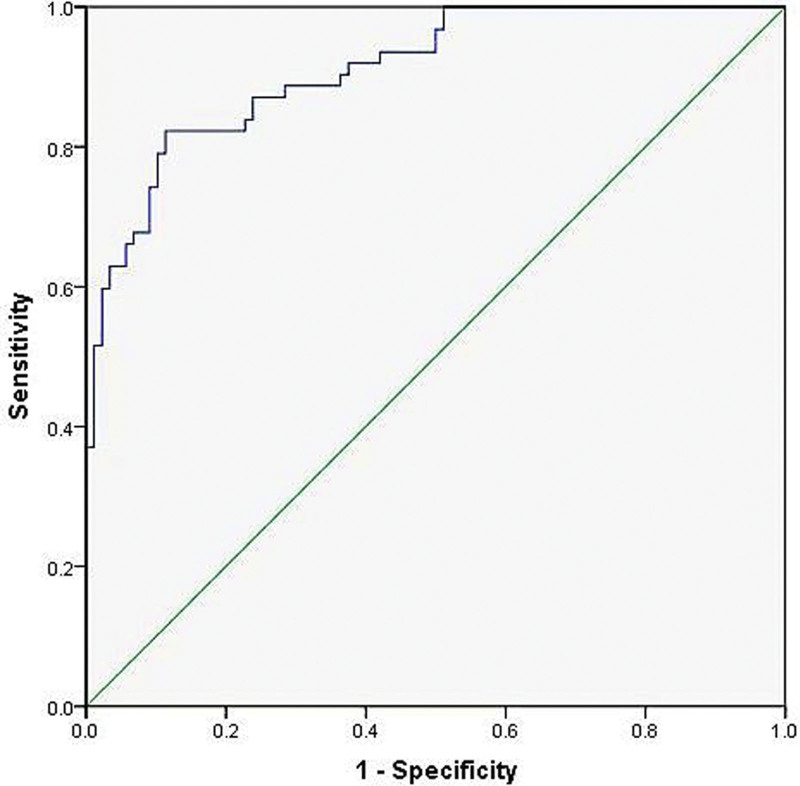
Diagnostic effects of internal validation of predictive models.

**Figure 6.**
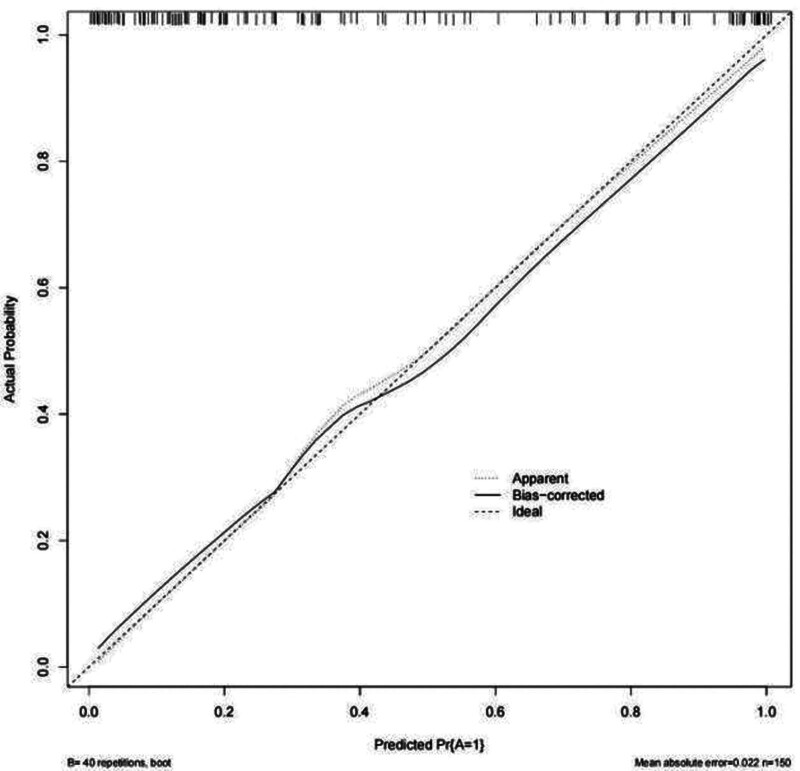
Internal validation calibration curve for AVF malfunction prediction model using Bootstrap method. AVF = arteriovenous fistula.

Using the bootstrap method with 1000 resamples for the internal calibration of the model, the results showed an AUC value of 0.911 (95% CI: 0.866–0.956). The sensitivity and specificity were 0.823 and 0.886, respectively. This indicates that the model maintained a high discriminatory capability (Fig. 5). The predictive curve for AVF dysfunction showed strong agreement with the actual curve (Fig. 6).

## 4. Discussion

In this study, we successfully established a predictive model for AVF dysfunction risk in patients undergoing MHD and performed internal validation along with calibration curve analysis. Through a retrospective analysis of clinical data from 150 patients undergoing MHD, we preliminarily identified 7 predictive factors closely associated with AVF dysfunction: advanced age, presence of diabetes, hyperlipidemia, D-D levels, blood phosphorus levels, female sex, and anastomotic width. Subsequent multivariate analysis revealed that advanced age, diabetes, hyperlipidemia, D-D, and blood phosphorus levels were independent predictive factors for AVF dysfunction. The prediction model established based on these factors showed high accuracy (AUC = 0.934) and reliability, offering clinicians a scientific and personalized approach to assess and predict AVF dysfunction risk in patients undergoing MHD, thereby enhancing treatment outcomes and quality of life.

A deeper examination of our results sheds light on the critical predictive factors for AVF dysfunction in patients with MHD and offers crucial insights for both clinical practice and medical research. First, we identified advanced age, diabetes, and hyperlipidemia as independent risk factors for AVF dysfunction. Our findings recognizing advanced age, diabetes, and hyperlipidemia as independent risk factors for AVF dysfunction are consistent with those of many previous studies.^[8–10]^ These factors are believed to be related to vascular function, structural changes, increased inflammatory responses, and vascular wall injuries, influencing AVF functionality and patency. These findings further validate the significant impact of these elements on AVF function in patients undergoing MHD. The prevalence of these factors in patients might be related to their overall health status, metabolic disturbances, and vascular injuries. Therefore, clinicians should pay special attention to the evaluation and management of AVF function in high-risk patients to minimize the occurrence of adverse events.

However, compared with previous research, our study identified several new predictive factors, including elevated levels of blood phosphorus and D-D. This highlights the pivotal role of hematological indicators in predicting AVF functional status. Previous studies have suggested that hyperphosphatemia may be correlated with AVF patency.^[11,12]^ Moon et al^[13]^ postulated that hyperphosphatemia (blood phosphorus > 5.5 mg/mL) is strongly positively correlated with the functional impairment of AVF within 1 year of post-maturation. Similarly, our findings showed that patients with hyperphosphatemia are more prone to AVF functional disturbances than those with normal levels, suggesting that hyperphosphatemia is an independent risk factor for AVF dysfunction. Continuous exploration of vascular access dysfunction has piqued interest in vascular calcification. Blood phosphorus is associated with vascular calcification. Persistent hyperphosphatemia can transform vascular smooth muscle cells into osteoblast-like cells,^[14]^ promoting the deposition of calcium and phosphorus on the inner vascular wall and leading to vascular calcification. In summary, elevated blood phosphorus levels may reflect mineral metabolic disturbances caused by renal function deterioration, which subsequently affect AVF function and vascular health.

The effect of D-D on AVF function remains controversial. Gumus^[15]^ reported that peripheral blood D-D concentrations < 0.63 are an independent predictor for maintaining AVF patency after surgical thrombectomy. However, Du et al^[16]^ noted that D-D (with each increase of 1 mg/L) was independently associated with AVF failure, with an odds ratio (OR value of 1.08 (95% CI: 1.02–1.15)). However, after adjusting for potential confounding factors, the D-dimer level (with each increase of 1 mg/L) was unrelated to AVF failure (OR = 1.06, 95% CI: 0.99–1.13). Our study indicates that an increase in peripheral blood D-D level is an independent risk factor for AVF dysfunction, consistent with the findings of Kong et al^[17]^ This might be attributed to D-D serving as a thrombus formation marker, and its elevation potentially reflects the extent of thrombus formation within the AVF vessel, thereby affecting AVF patency and function. Effective anticoagulation therapy may lower peripheral blood D-D concentrations and reduce thrombus formation, decreasing AVF dysfunction incidence. These insights offer new clues and avenues for a deeper comprehension of the mechanisms underlying AVF functional abnormalities.

Additionally, the univariate analysis indicated a relationship between anastomosis width and AVF dysfunction. The width of the anastomosis, an essential parameter in AVF surgical procedures, may correlate with factors such as surgical success rate, blood flow rate, and vascular pressure, subsequently influencing the functional status of the AVF.^[18,19]^ Previous studies have reported that a radial artery diameter < 2.1 mm is an independent risk factor for AVF dysfunction.^[20]^ However, the logistic regression analysis results in our study did not corroborate preoperative vascular diameter and anastomotic width as independent risk factors for AVF dysfunction. This may be attributed to our center’s preliminary screening of preoperative vascular diameters and enhancements in surgical techniques.

This study has several limitations that warrant consideration. First, patients with imaging-detected upper limb arterial or venous narrowing were not subjected to AVF surgery but instead received permanent catheterization or other treatments, which may have resulted in patient selection bias. Second, our sample was derived solely from a specific hospital population and possessed restricted geographical and demographic characteristics, which may hinder the generalizability of our findings. Hence, multicenter and multiregional prospective studies would provide more robust evidence to validate and extend our results. Furthermore, this study only selected basic clinical and hematological markers as predictors, and it is plausible that other latent factors were not considered. For instance, genetic variables, inflammatory markers, and vascular functionality assessments could impact AVF dysfunction but were not meticulously analyzed in this study. Future research could expand the scope of these factors and employ a more comprehensive set of indicators to evaluate the functional predictability of AVFs. Finally, although we proposed a model predicting AVF dysfunction and performed internal validation, external validation remains necessary to affirm the model’s accuracy and reliability. Validating our model across several independent samples further solidified and enhanced its predictive performance. We plan to address these constraints and shortcomings in future studies by implementing appropriate rectifications. By integrating more comprehensive metrics, larger sample sizes, and stringent research designs, we aimed to delve deeper into the mechanisms underlying AVF dysfunction, offering a more precise and personalized predictive model to enhance treatment outcomes and quality of life in MHD patients.

## 5. Conclusion

In summary, this study offers pivotal insights into predicting AVF dysfunction in patients by constructing and validating a risk model. Our study identified advanced age, concurrent diabetes, hyperlipidemia, elevated D-dimer levels, and hyperphosphatemia as independent risk factors for AVF dysfunction in patients undergoing MHD. Drawing on these variables, we successfully constructed a predictive model demonstrating robust accuracy and reliability after internal validation. These findings have significant implications in clinical practice and medical research. Primarily, the model can assist clinicians in the early evaluation of the risk of AVF dysfunction in patients undergoing MHD, allowing the formulation of pertinent preventative and interventional strategies. Moreover, it provides physicians with an evidence-based foundation for devising personalized treatment regimens to enhance dialysis outcomes and quality of life in MHD patients. Additionally, the outcomes of this research will pave the way for innovative approaches and methodologies in related studies, laying the groundwork for a deeper exploration of the mechanisms and factors associated with AVF dysfunction.

## Acknowledgments

We would like to thank Editage (www.editage.cn) for English language editing.

## Author contributions

**Conceptualization:** Liqin Wang, Yanna Yang.

**Data curation:** Liqin Wang, Yanna Yang.

**Formal analysis:** Liqin Wang, Yanna Yang.

**Investigation:** Liqin Wang, Qianqian Zhao.

**Methodology:** Liqin Wang, Qianqian Zhao.

**Validation:** Qianqian Zhao.

**Visualization:** Qianqian Zhao.

**Writing – original draft:** Liqin Wang, Yanna Yang.

**Writing – review & editing:** Liqin Wang, Yanna Yang.

## References

[1] Basile C Davenport A Mitra S. Frontiers in hemodialysis: innovations and technological advances. Artif Organs. 2021;45:175–82. 32780472 10.1111/aor.13798

[2] Wei S Jiao J Yu Y. Long-term arteriovenous fistula prognosis for maintenance hemodialysis patients who accepted PIRRT by using arteriovenous fistula. Int J Artif Organs. 2023;46:195–201. 36945121 10.1177/03913988231162384

[3] Li Y Cui W Wang J. Comparative efficacy of five balloons for treating autogenous arteriovenous fistula stenosis: a Bayesian network meta-analysis. Ann Palliat Med. 2022;11:2574–85. 35610193 10.21037/apm-21-2898

[4] Romyn A Rush KL Hole R. Vascular access transition: experiences of patients on hemodialysis. Nephrol Nurs J. 2015;42:445–53; quiz 454. 26591269

[5] Meola M Marciello A Di Salle G. Ultrasound evaluation of access complications: thrombosis, aneurysms, pseudoaneurysms and infections. J Vasc Access. 2021;22:71–83. 34313154 10.1177/11297298211018062 PMC8607320

[6] Li Y Cui W Wang J. Factors associated with dysfunction of autogenous arteriovenous fistula in patients with maintenance hemodialysis: a retrospective study. Ann Palliat Med. 2021;10:4047–54. 33832310 10.21037/apm-20-2196

[7] Rogers S Simm K McCollum C. Arteriovenous fistula surveillance using tomographic 3D ultrasound. Eur J Vasc Endovasc Surg. 2021;62:82–8. 33896727 10.1016/j.ejvs.2021.03.007

[8] Wei S Liu N Fu Y. Novel insights into modifiable risk factors for arteriovenous fistula failure and the importance of CKD lipid profile: a meta-analysis. J Vasc Access. 2023;23:11297298221115557. 10.1177/11297298221115557 36951426

[9] Chytilova E Jemcov T Malik J. Role of Doppler ultrasonography in the evaluation of hemodialysis arteriovenous access maturation and influencing factors. J Vasc Access. 2021;22:42–55. 10.1177/1129729820965064 PMC8607314 34281411

[10] Luo Q Liu H Yang Q. Analysis of factors influencing restenosis after percutaneous transluminal angioplasty. Blood Purif. 2022;51:1031–8. 35504252 10.1159/000524159

[11] Zhou M Lu FP. Effect of hyperphosphatemia on patency rate of arteriovenous fistula of patients with late fistula dysfunction/failure after reoperation. Zhonghua Yi Xue Za Zhi. 2018;98:3406–10. 30440134 10.3760/cma.j.issn.0376-2491.2018.42.006

[12] Li Y Cui W Wang J. Factors associated with dysfunction of autogenous arteriovenous fistula in patients with maintenance hemodialysis: a retrospective study. Ann Palliat Med. 2021;10:4047–54. 33832310 10.21037/apm-20-2196

[13] Moon JY Lee HM Lee SH. Hyperphosphatemia is associated with patency loss of arteriovenous fistula after 1 year of hemodialysis. Kidney Res Clin Pract. 2015;34:41–6. 26484018 10.1016/j.krcp.2015.02.001 PMC4570653

[14] Shimokado A Sun Y Nakanishi M. Smad3 plays an inhibitory role in phosphate-induced vascular smooth muscle cell calcification. Exp Mol Pathol. 2014;97:458–64. 25303897 10.1016/j.yexmp.2014.10.005

[15] Gumus F. Patency rates after successful arteriovenous fistula thrombectomy: relevance of the Flow/d-Dimer ratio in the decision-making. Vasc Endovascular Surg. 2020;54:670–5. 32720863 10.1177/1538574420945064

[16] Du J Kong X Liang L. Plasma D-Dimer level and the failure of forearm autologous arteriovenous fistula in patients with end-stage renal disease. Ther Apher Dial. 2020;24:400–7. 31705787 10.1111/1744-9987.13454

[17] Kong X Tang L Liang L. Clinical outcomes following the surgery of new autologous arteriovenous fistulas proximal to the failed ones in end-stage renal disease patients: a retrospective cohort study. Ren Fail. 2019;41:1036–44. 31814501 10.1080/0886022X.2019.1696210 PMC6913653

[18] Zhou M Chen G Feng L. Effects of ultrasound-guided percutaneous transluminal angioplasty for stenosis of arteriovenous fistula used for hemodialysis and related factors influencing patency. Ann Ital Chir. 2020;91:55–60. 32180566

[19] Patel P Prabha V Verneker RR. Role of color Doppler assessment in predicting outcomes of wrist Brescia-Cimino arteriovenous fistula creation: a single-center prospective study. Indian J Urol. 2023;39:33–8. 36824103 10.4103/iju.iju_190_22 PMC9942221

[20] Misskey J Hamidizadeh R Faulds J. Influence of artery and vein diameters on autogenous arteriovenous access patency. J Vasc Surg. 2020;71:158–72.e1. 31303475 10.1016/j.jvs.2019.03.075

